# Mixed-dust pneumoconiosis in a dental technician: a multidisciplinary diagnosis case report

**DOI:** 10.1186/s12890-022-01948-6

**Published:** 2022-04-27

**Authors:** Luigi Di Lorenzo, Francesco Inchingolo, Antonella Pipoli, Filippo Cassano, Maria Elena Maggiore, Angelo Michele Inchingolo, Sabino Ceci, Assunta Patano, Giuseppina Malcangi, Antonio Mancini, Giosi Longo, Rossella Attimonelli, Eugenio Maiorano, Rocco Laviano, Nicola Mariano Manghisi, Antonio Scarano, Felice Lorusso, Antonio Di Lorenzo, Alessio Danilo Inchingolo, Gianna Dipalma

**Affiliations:** 1 Department of Interdisciplinary Medicine, University of Medicine Aldo Moro, 70124 Bari, Italy; 2 National Institute for Insurance Against Injuries at Work (INAIL), Bari, Italy; 3 Department of Emergency and Transplantation, Pathology Section, University of Bari, Bari, Italy; 4 Department of Earth and Geoenvironmental Sciences, University of Bari, Bari, Italy; 5 Department of Prevention, Prevention and Safety at Work Service, Local Health Board of Brindisi, Brindisi, Italy; 6 Department of Innovative Technologies in Medicine and Dentistry, University of Chieti-Pescara, 66100 Chieti, Italy; 7 Department of Biomedical Science and Human Oncology, University of Bari, 70124 Bari, Italy

**Keywords:** Pneumoconiosis, Dental technician, Environmental analysis, Mineralogical analysis, Histologic analysis

## Abstract

**Background:**

In dental laboratories, exposure to crystalline silica can occur during procedures that generate suspended mineral dusts, e.g. dispersion of mixing powders, removal of castings from molds grinding, polishing of castings and porcelain, and use of silica sand for blasting. There is also a large list of toxic agents (acrylic resins, polymeric materials, etc.) used to produce removable and fixed prostheses, but also impression materials and more. Using personal protective equipment and other aids reduces the exposure to these potentially harmful agents.

**Case presentation:**

We report the case of a 42-year-old male dental technician who began to suffer from a dry cough and exertional dyspnea after approximately 15 years of work. The operations he conducted for his job resulted in the generation of crystalline silica, aluminum, chromium and titanium dust. The worker did not regularly wear personal protective equipment and some of the above operations were not carried out in closed circuit systems. The Chest X-ray showed diffused micronodules in the pulmonary interstitium of the upper-middle lobes, bilaterally, and a modest left basal pleural effusion. Simple spirometry showed small airway obstruction in its initial stage. High Resolution Computerized Tomography of the chest showed bilateral micronodulation of a miliariform type, with greater profusion to the upper lobes, also present in the visceral pleura, bilaterally. Histological examination showed aggregates of pigment-laden macrophages forming perivascular macules or arranged in a radial pattern around a core of sclerohyalinosis. Scanning Electron Microscopy and Energy Dispersive Spectrometry revealed several mineral particles, typically characterized by the presence of crystalline silica and metal aggregates. The environmental concentrations of total dust and its respirable fraction were all lower than the relative TLV-TWA—ACGIH, yet not negligible.

**Conclusions:**

The above findings and a multidisciplinary assessment led to the diagnosis of mixed dust pneumoconiosis s/q with 2/2 profusion of occupational origin. This diagnosis in a dental technician was supported for the first time in literature by environmental exposure analysis.

## Background

Traditionally, dentures are produced with a metallic infrastructure and a resin veneering layer, according to the "lost wax" technique. In dentistry, a wide range of chemical agents are used, such as eugenol-containing materials, alloys, polymer materials, acrylic resins, cements, ceramics, sealers, hypochlorite, waxes, and impression materials. The exposure to these materials can lead to health problems [1]. Methacrylate monomers are frequently present in dental laboratories and their use can cause a wide range of adverse health effects, such as skin, eyes, and mucous irritation, allergic dermatitis, asthma attacks, and neurological symptoms [2, 3]. Indeed, contact dermatitis to acrylates is very frequent among laboratory technicians and dentists due to close contact with these materials during the manufacturing and implantation of dental prosthesis [4, 5].

In particular, cobalt-chrome and molybdenum alloys are used for creating metal structures and refractory materials to make metal skeleton mold. Refractory materials are composed of quartz, one of the most frequent forms of crystalline silica found in nature [5, 6].

Chronic inhalation of mixed mineral dusts containing respirable particles of crystalline silica and metals produced during manufacturing processes can cause a type of pneumoconiosis referred as “mixed mineral dust”. Indeed, the term Mixed Dusts Pneumoconiosis (MDP) has been used since the middle of the last century to describe certain forms of pneumoconiosis in foundry workers, who are simultaneously exposed to crystalline silica and to other less or non-fibrous mineral dusts. These present highly variable histological, functional and radiological pictures, and are sometimes difficult to define nosologically [7–10].

The pathogenetic mechanism triggered by the deposition of free crystalline silica particles is characterised by injury to the first type of pneumocytes by a predominantly direct and indirect oxidative process, the latter being mediated by the activation of pulmonary inflammatory cells, in particular macrophages and neutrophils. Free crystalline silica particles and pro-inflammatory cytokines can thus penetrate the pulmonary interstitium, both of which can activate lymphocytes, fibroblasts and myofibroblasts, resulting in interstitial fibrosis of a mainly nodular type.

In many occupational activities the mineral dusts produced may consist, in addition to crystalline silica, of non-fibrous silicates containing varying amounts of iron oxide and other minerals. These mineral dusts are called mixed dusts, and the different minerals present, together with crystalline silica, can cause multiform histological lesions of the lung interstitium. This can lead to the formation of dust macules, classic silica nodules and/or fibrotic nodules, and even massive fibrotic lesions, which characterise the histological picture of so-called MDP. [11]

Many cases of MDP in dental technicians due to chronic inhalation of crystalline silica, silicon carbide, asbestos, cobalt, molybdenum beryllium and nickel dusts, have been described in literature [12–22]. Particularly in the past, these workers were exposed to inhalation of mineral dusts, including crystalline silica (SiO_2_), in poorly ventilated and unsuitable extraction systems [12–22]*.* A recent study, conducted in Turkey, on 893 dental technicians admitted to Ankara hospital from January 2007 until May 2012, found 90 cases of pneumoconiosis with a prevalence of 10.1% [23].

Furthermore, a recent Italian study reports the appearance of signs and symptoms of MDP in a dental technician after only two years of working in a dental laboratory where ceramic and metal dusts are present [24].

Environmental monitoring of the air in the workplace should therefore be carried out in order to assess the total dust concentration and its composition, focusing on the concentration of crystalline silica.

In addition, it would be necessary to measure the concentration of potentially pneumoconiogenic mineral dust in both inhalable (mean aerodynamic mass diameter (MAAD) < 100 µm) and respirable (MAAD < 10 µm) fractions of the total ambient dust, in order to better protect the health of dental laboratory workers [25].

## Case presentation

The present investigation was conducted in University of Bari (Italy), in accordance with ethical principles, including the World Medical Association Declaration of Helsinki and the additional requirements of Italian law. The participant signed an informed consent to the treatment. We report the case of a 42-year-old Italian man E.S., (height 1.68 m, weight 89 kg), a former smoker who had smoked 20 cigarettes a day from the age of 20 until the age of 34 (14 years) and had worked in a dental laboratory for 15 years. When he came to our observation, he reported that during the previous 3 months he had developed a dry and persistent cough and dyspnoea that occured after 400–500 m of walking on level ground, in the absence of other clinically evident cardio-respiratory pathologies.

**Fig. 1:**
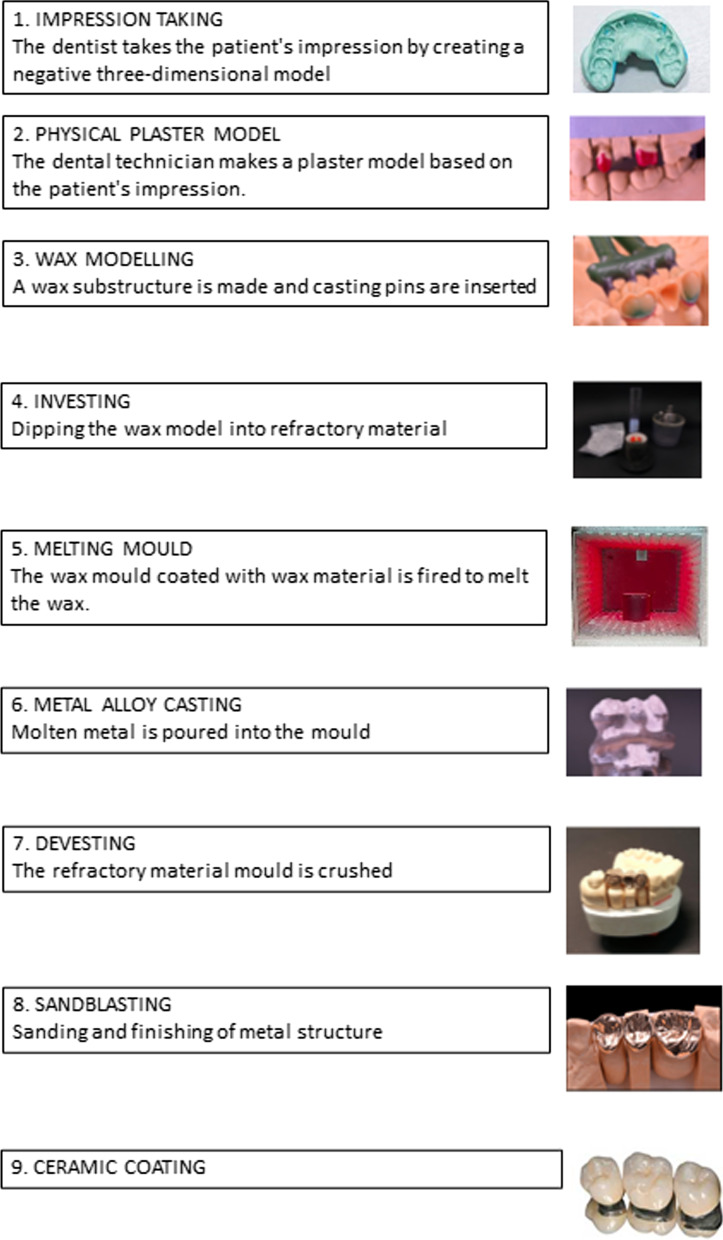
Production of dental prostheses using the “lost wax” technique

The dental laboratory in which this technician had worked produced mobile skeletal dental prostheses. E.S. was engaged in the production of a metal skeleton, suitably coated with resin of a similar colour as gums, which functions as a support for artificial teeth. The production process of the prostheses according to the "lost wax" technique is shown in Fig. 1. During the devesting phase (Fig. 1, point 7), the refractory material mold was removed and quartz-containing dust was formed. The operations were carried out in the absence of extraction systems and the worker reported that he did not regularly wear the personal protective equipment (PPE) provided by his employer. The sandblasting phase (Fig. 1, point 8), which involved the use of aluminium oxide sand, was carried out in a closed automatic sandblasting machine.

**Fig. 2:**
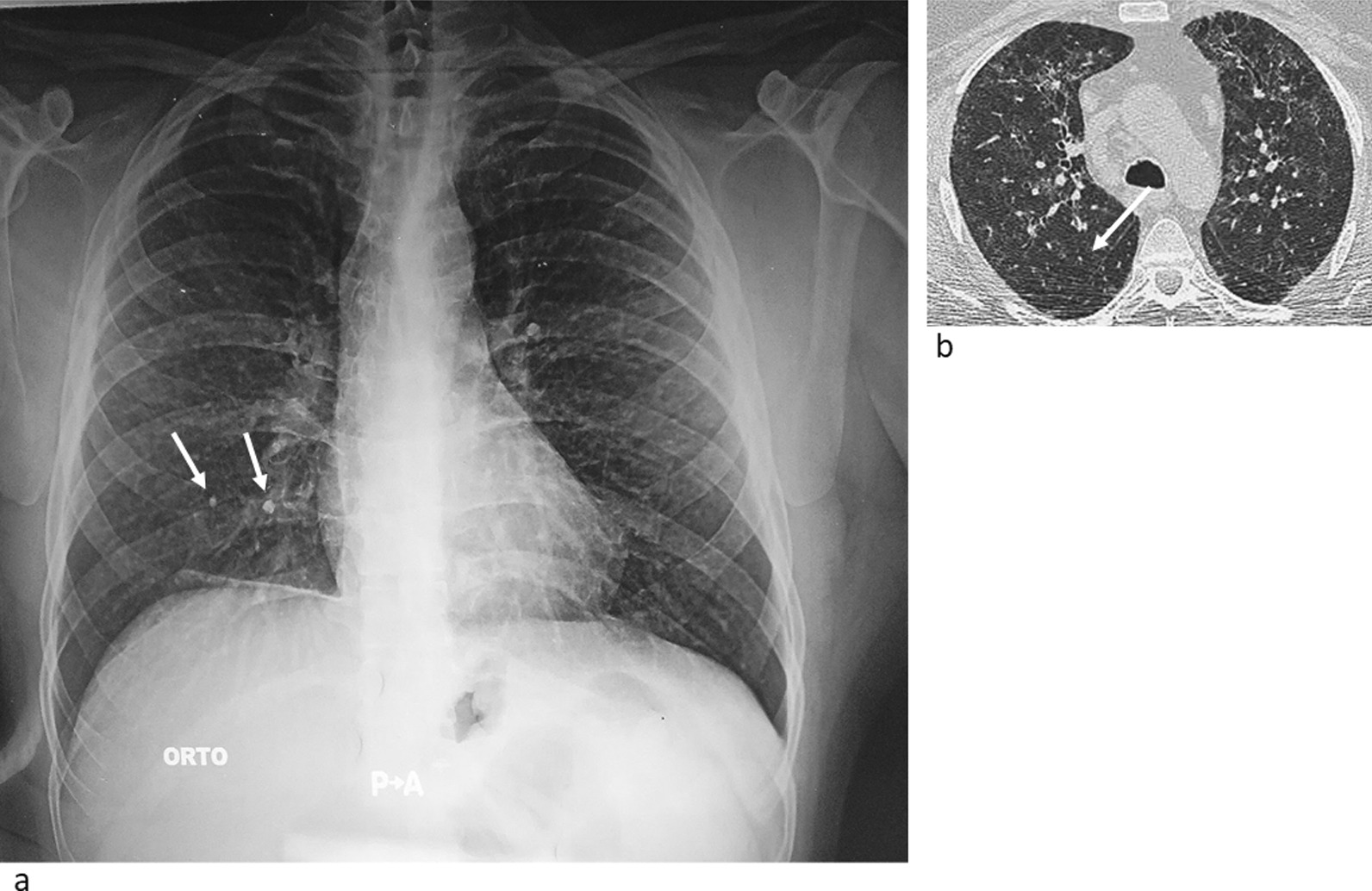
**a** Chest X-ray showing radiopacity in the mid-apical fields and fibrotic findings **b** Chest High Resolution Computerized Tomography (HRCT) showing miliary micronodulation, bilaterally

For the above-mentioned respiratory symptoms, the laboratory occupational physician had requested a chest X-ray to be performed at the Unit of Occupational Medicine of the University Hospital Polyclinic of Bari (Italy). The radiogram was read according to the National Institute for Occupational Safety and Health (NIOSH) guidelines [26]. The presence of small rounded (q) and, above all, irregular (s) radio-opacities was detected, found mainly in the mid-apical sections of the lungs, with 2/1 profusion of parenchymal fibrotic outcomes in the anterior-inferior segment of the right basal bronchus, with a partially calcified fibrotic stria on the right side of the pulmonary base and a "frontal" calcified diaphragmatic pleural plaque on the right side (Fig. 2a). For further investigations, the subject was admitted to the Thoracic Surgery Unit of the University Hospital Polyclinic of Bari (Italy). Simple spirometry only showed initial obstruction of the small airways (FVC: 3.98L 94% of predicted; FEV1: 3.00L 85%, FEV1/FVC: 76%; FEF 25%: 9.34L/s 125%; FEF 50%: 3.22 L/s 68%; FEF 75%: 0.65 L/s 33%; PEF: 9.55 L/s 110%). The lung diffusing capacity for Carbon Monoxide (DLCO), with an increased DLCO to an alveolar ventilation (AV) ratio of 24%.

To assess the amount of exertion causing dyspnoea, the 6-min walk test (6MWT) was performed [27], in a corridor of our department 30 m long. At the beginning and end of the test, oxygen saturation (SaO_2_) was measured with a digital saturation meter certified according to Directive 94/42/EEC [28], The SaO_2_ at the beginning of the test was 97%. The intensity of fatigue and dyspnoea were assessed using the modified Borg scale 0–10 (mBS) [29].

In order to assess the impact of the pathology on the distance covered in 6 min, the maximum theoretical distance that a man with the age, height and weight of the worker in the study could be expected to walk was calculated according to the equation proposed by Enright et. al. This distance was 595.3 m [30].

At the end of the test the worker walked 520 m and his SaO_2_ was 95% and he reported a fatigue of 3/10 (moderate) and dyspnoea of 3/10 (moderate-strong) according to the mBS 0–10 [29].

Chest High-Resolution Computed Tomography (HRCT), evaluated according to the International Classification of HRTC for Occupational and Environmental Respiratory Diseases [31], confirmed the bilateral micronodulation of miliariform type, with greater profusion to the upper lobes, also present in the visceral pleura, bilaterally. It also showed a small parenchymal thickening at the base of the left lung and some enlarged lymph nodes in the para-aortic, precarenal and subcarenal areas (Fig. 2b).

**Fig. 3:**
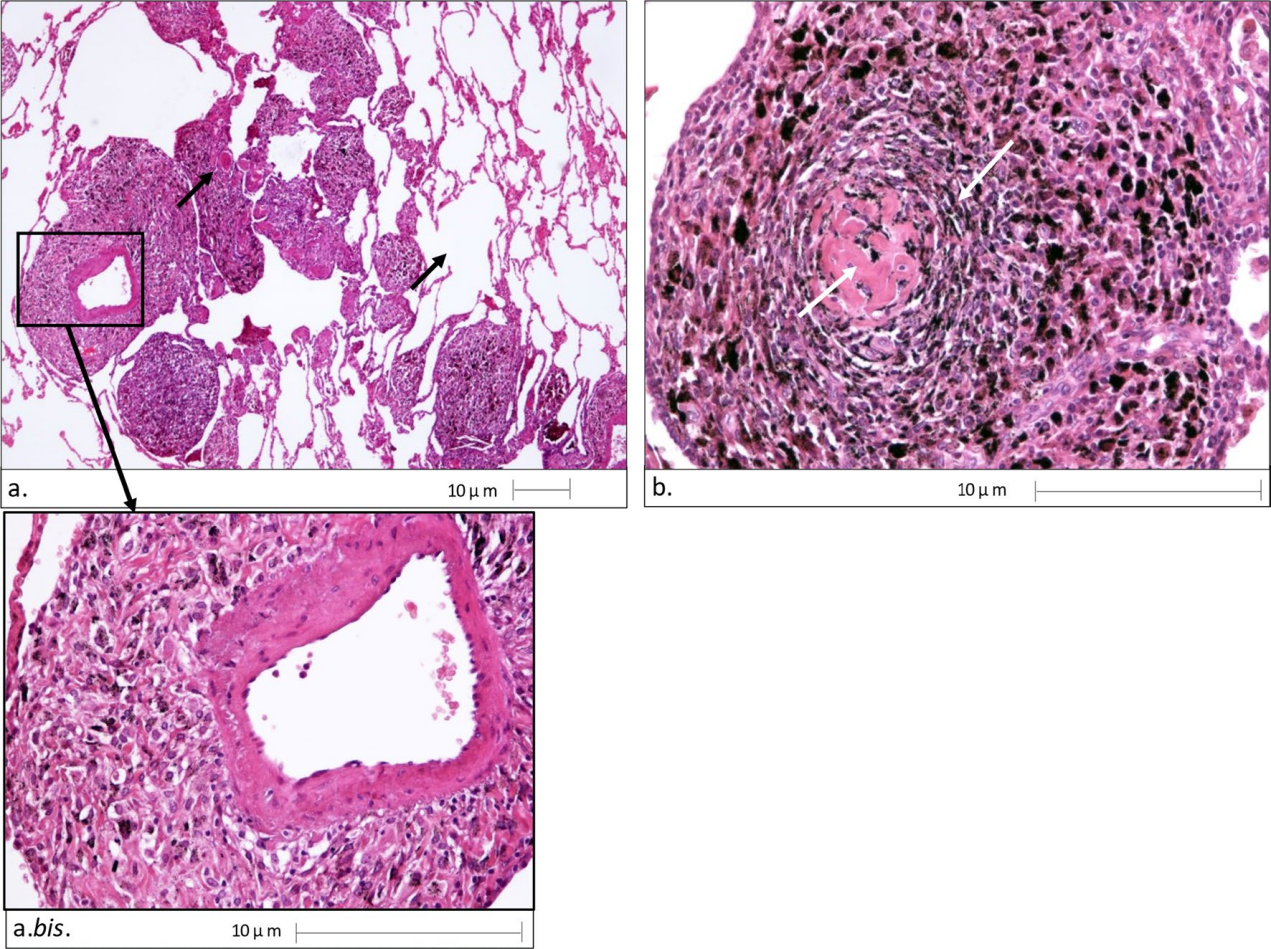
Photomicrographs of histological image, Haematoxylin Eosin staining, produced by Nikon Eclipse 80i microscope equipped with Digital camera Nikon DS-Fil: **a** 40 × magnification (scale bar 10 µm) shows granulomatous lesions in a context of centrolobular emphysema, particularly noticeable: gigantocellular cells, macula formed by perivascular interstitial aggregates of macrophages loaded with intracytoplasmic dust pigment material; a.bis. 200× magnification of perivascular macula; **b** Magnification 200×, showing fibrotic mixed dust lesions characterised by interstitial aggregates of macrophages laden with intracytoplasmic dust pigment arranged in a radial manner with respect to a central core of scleroialinosis

The surgeons performed an exploratory thoracotomy with pleural-parenchymal biopsy. Histological examination showed granulomatous lesions in a context of centrilobular emphysema. Particularly noticeable were gigantocellular cells, and macula formed by perivascular interstitial aggregates of macrophages loaded with intracytoplasmic dust pigment material. In addition, fibrotic mixed dust lesions were visible, characterized by interstitial aggregates of intracytoplasmic dust pigment-laden macrophages, arranged in a radial manner in relation to a central core of sclerohyalinosis (Figs. 3).

Therefore, in order to better define the etiological origin of the pneumoconiosis, after obtaining the worker’s informed consent, the occupational physicians requested the remaining biopsy specimen of the base of the left lung in order to carry out Scanning Electron Microscopy (SEM) [32].

**Fig. 4:**
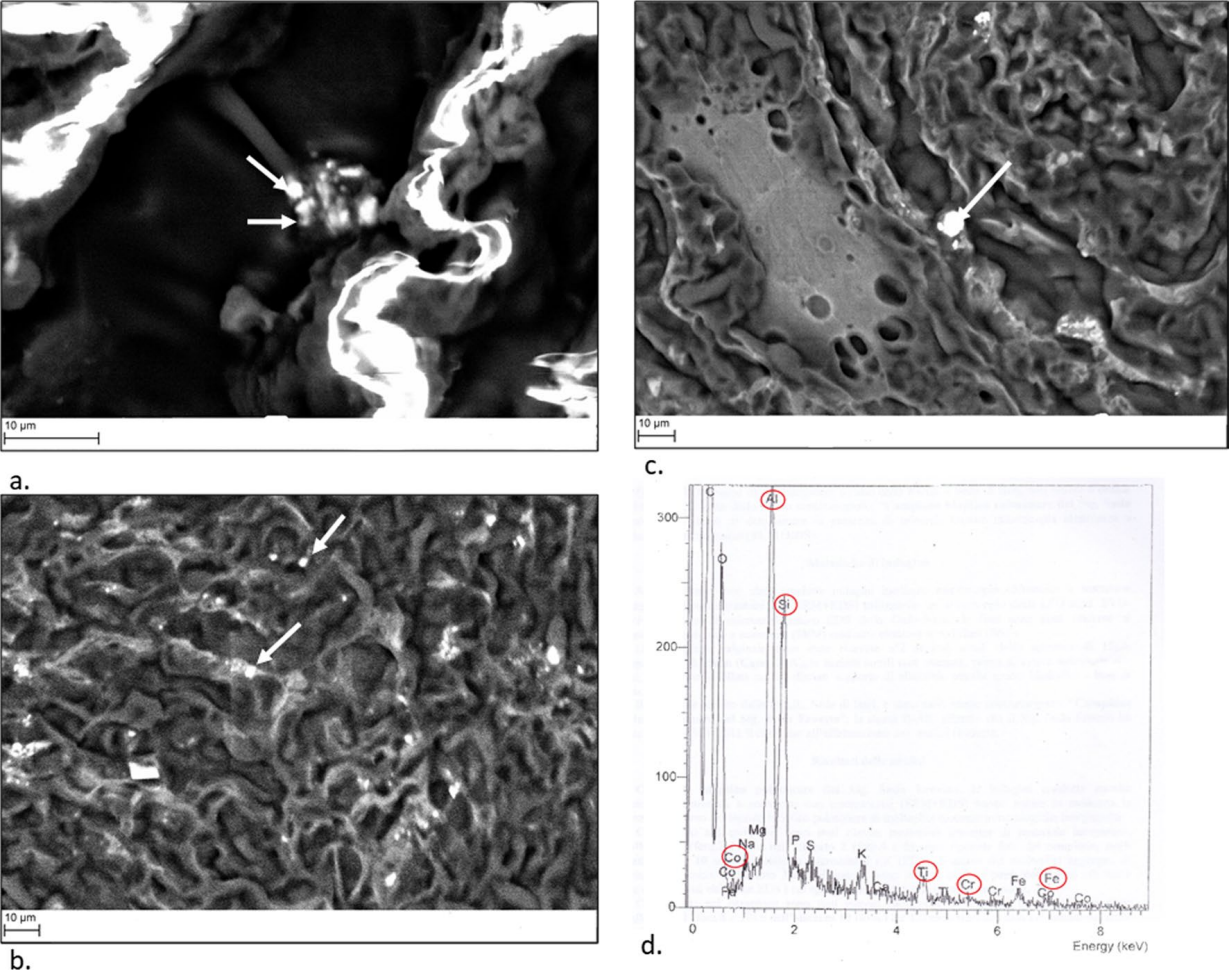
Photos of tissue sections examined (**a**, **b**, **c**) and Energy Dispersive Spectrometer (EDS) plotting of element peaks (**d**) produced by Scanning Electron Microscope (SEM), model EVO50XVP by LEO, equipped with Energy Dispersive Spectrometer (EDS), with Oxford Silicon drift X-max detector (80 mm^2^) equipped with Super ATW © (Super Atmosphere Thin Window)

The SEM revealed several mineral particles, whose composition (Figs. 4a–c) was identified using an Energy Dispersive Spectrometer (EDS). The presence of multiple aggregates of silicates, crystalline silica, aluminium (Al), iron (Fe), chromium (Cr), cobalt (Co) and titanium (Ti) was detected (Fig. 4d).

We requested and obtained the Occupational Risk Assessment Document of the dental laboratory, which contained the safety data sheets of the substances used. These sheets documented the presence of quartz, cristobalite and magnesium oxide in the refractory material used in the investing phase (Fig. 1 point 4) and the presence of chromium, cobalt and molybdenum in the metal alloy used in the casting phase (Fig. 1 point 6). Moreover, in the same document, there were the results of the environmental analysis, carried out by means of constant flow sampling, four at a fixed location in the ceramics room, the plaster room, the sandblasting room and the finishing room, and two by means of personal samplers placed on the plaster preparer and sandblaster’s lab coats. Each sample was taken for eight hours, continuously. The amount of mineral dusts was determined by double weighting method using an electronic microbalance [11].

The environmental concentrations of the inhalable fraction, ranging from 0.88 to 1.90 mg/m^3^, and its respirable fraction, ranging from 0.18 to 0.55 mg/m^3^, measured by fixed sampling at different workstations, were all below the relevant Threshold Limit Values-Time Weighted Average (TLV-TWA) proposed by the American Conference of Governmental Industrial Hygienists (ACGIH) [33] (Table 1).

**Table 1:** Concentration of inhalable fraction and respirable fraction, obtained by stationary and individual samplers, in the workplace

Sampling method	Sampling site	Inhalable fraction^a^ (mg/m^3^)	Respirable fraction ^b^ (mg/m^3^)
Stationary	Ceramic room	0.88	0.51
	Plaster room	1.90	0.49
	Sandblasting room	1.76	0.55
	Finishing room	0.86	0.18
Individual	Plaster preparation worker	1.55	0.60
	Sandblasting worker	1.41	0.82

TLV-TWA (Threshold Limit Value—Time Weighted Average) by the American Conference of Governmental Industrial Hygienists

^a^Inhalable fraction = 10 mg/m^3^

^b^Respirable Fraction = 3 mg/m^3^

The two concentrations of total dust, 1.41 and 1.55 mg/m^3^, and of its respirable fraction, 0.60 and 0.82 mg/m^3^, measured by individual sampling, were also all below the relevant TLV-TWAs [33] (Table 1).

Based on the clinical, radiological, histological, mineralogical and toxicological findings available, the diagnosis of MDP s/q was established, with 2/2 profusion, predominantly affecting bilaterally the parenchyma of the mid-apical lung segments, with a slight functional impairment [7].

After the diagnosis, the worker was judged not suitable for his specific job for six months, also to allow him to undergo pharmacological and rehabilitation therapy.

The drug therapy consisted of prednisone per os 25 mg at 08:00 a.m. and 5 mg at 03:00 p.m. for seven days, then 25 mg at 08:00 a.m. for seven days and finally 12.5 mg at 08:00 a.m. for another seven days. In addition, after the diagnosis, the patient also underwent a course of aerosol therapy with beclomethasone dipropionate 0.8 mg and salbutamol 1.6 µg, every eight hours, for 60 days. The aerosol therapy was carried out using a diaphragmatic ventilation technique, which was taught to the subject by an experienced physiotherapist, to optimise the penetration of the drugs and their deposition even in the most distal airways.

The worker underwent another medical examination after six months, during which he reported an improvement in his general health, the disappearance of cough and a reduction in the subjective sensation of dyspnoea on exertion. Functional tests were repeated. The worker showed an improvement in spirometry values (FVC: 4.25 L 100% of predicted; FEV1: 3.52 L 91%; FEV1/FVC: 75%; FEF 25%: 8.47 L/s 113%; FEF 50%: 4.49 L/s 95%; FEF 75%: 0.64 L/s 33%) and in 6MWT (SaO_2_ before the test 98%, SaO_2_ after the test 97%, distance covered 580 m, 60 m more than the first 6MWT, perceived fatigue 2/10, perceived dyspnoea 2/10).

In view of the improvement in the functional tests, it was not considered appropriate to repeat the radiological examinations to avoid further exposure to ionising radiation for the worker.

In view of the improvement in clinical conditions and respiratory function tests, the worker was judged by the occupational physician to be suitable for work as a dental technician, with the exclusion of performing devesting phase operations and with a prescription to avoid entering the work environments in which these operations are carried out and the obligation to use appropriate personal respiratory protective equipment in case of occasional formation of bronchoirritant aerosols.

## Discussion and conclusions

For the first time in literature, the results of an environmental survey carried out in a dental laboratory are reported. This identified the low but non-negligible concentrations of pneumoconiogenic minerals and metals in both inhalable and respirable fraction of total dust in working environment. These dusts corresponded to the raw materials and metals used in the production of skeletal dental prostheses. Their toxicological data sheets were included in the laboratory's occupational risk assessment document. The mineralogical examination confirmed the presence of inhaled mineral particles derived from raw materials in the pulmonary biopsy fragments of the worker.

Clinical data, functional respiratory examination and, above all, histological examination and imaging, evaluated according to NIOSH guidelines, made it possible to give further biological plausibility to the results of the toxicological investigations carried out on the environment and lung tissue.

Therefore, this is the first study that has been able to demonstrate a causal link between occupational exposure to pneumoconiogenic mineral and metal dusts, in the absence of appropriate extraction systems, and MDP in a dental technician who did not regularly use personal respiratory protective equipment.

Even in sectors that are not "traditionally" considered to be at risk of MDP, there may be particular environmental situations and/or subjective behaviour on the part of workers that can lead to levels of exposure to mineral dusts able to favour the onset of lung parenchyma diseases. Therefore, this paper confirms that employers and occupational physicians must commit themselves to carrying out primary and secondary prevention interventions in all production activities that may lead to exposure of workers to pneumoconiogenic mineral dusts.

The case described shows that good industrial hygiene and occupational medicine practices and collaboration among specialists from different medical and mineralogical disciplines, provided the necessary documentation to establish the causal link between a dental technician's occupational exposure to respirable mineral dusts, the onset of an uncommon MDP to readmit this dental technician to work, after appropriate treatment and with the aforementioned exclusions and prescriptions.

For preventive purposes, however, it is necessary to remember that employers of dental laboratories must always engage in primary and secondary prevention [34] after appropriate training and information. Therefore, the reduction of the respiratory risk in dental laboratories should also favor the choice of less toxic materials, the use of dust extraction and room ventilation systems and Computer Aided Design—Computer Aided Manufacturing (CAD-CAM) technologies for the fabrication of prostheses [34, 35].

## Data Availability

All experimental data to support the findings of this study are available contacting the corresponding author upon request. The authors have annotated the entire data building process and empirical techniques presented in the paper.

## Abbreviations

ACGIH: American Conference of Governmental Industrial Hygienists
AV: Alveolar ventilation
CAD-CAM: Computer-Aided Design-Computer-Aided Manufacturing
DLCO: Lung diffusing capacity for Carbon Monoxide
EDS: Energy Dispersive Spectrometer
FEF: Forced expiratory flow
FEV1: Forced expiratory volume in the 1st second
FVC: Forced vital capacity
HRCT: High-Resolution Computed Tomography
mBS: Modified Borg Scale
MDP: Mixed Dusts Pneumoconiosis
MAAD: Mass average aerodynamic diameter
NIOSH: National Institute for Occupational Safety and Health
PMMA: Poly-methyl-methacrylate
PEF: Peak expiratory flow
PPE: Personal protective equipment
SaO_2_: Oxygen saturation
SEM: Scanning Electron Microscopy
TLV-TWA: Threshold Limit Values-Time Weighted Average
6MWT: 6 Minutes walking test
